# Frailty in elderly with a rare genetic disease: a geriatric score analysis in C1 inhibitor Hereditary Angioedema

**DOI:** 10.3389/fmed.2026.1834045

**Published:** 2026-06-26

**Authors:** Paola Triggianese, Francesca Cedola, Giulio Barone, Enrica Marchionni, Liliana Mannucci, Alessandra Valentini, Sergio Bernardini, Giuseppe Novelli, David Della-Morte

**Affiliations:** 1 Department of Biomedicine and Prevention, University of Rome Tor Vergata, Rome, Italy; 2 Hereditary and Acquired Angioedema Reference Center, Department of Scienze Mediche, Tor Vergata University Hospital, Rome, Italy; 3 Nutrition and Geriatrics, Tor Vergata University Hospital, Rome, Italy; 4 Medical Genetics Unit, Tor Vergata University Hospital, Rome, Italy; 5 Department of Experimental Medicine and Surgery, Tor Vergata University Hospital, Rome, Italy; 6 Department of Neurology, Evelyn F. McKnight Brain Institute, Miller School of Medicine, University of Miami, Miami, FL, United States

**Keywords:** aging, elderly, frailty, Hereditary Angioedema, quality of life, rare disease

## Abstract

**Introduction:**

Patients with Hereditary Angioedema related to deficiency in C1-esterase inhibitor (HAE-C1INH) experience a relevant burden of both lifelong disease and treatments. The coexistence of additional comorbidities concurs with the reduction of the quality of life (QoL) often resulting in social-psychological isolation and potential multidimensional frailty. We aimed at assessing for the first time frailty in elderly with HAE-C1INH by using a comprehensive geriatric assessment (CGA).

**Patients and methods:**

A single-center cross-sectional comparative study with a control group included elderly patients with a defined diagnosis of HAE-C1INH recruited from the HAE Reference Center (Tor Vergata University Hospital, Rome, Italy). Healthy controls (HC) were enrolled from subjects referring to the Geriatric Unit, at the same Hospital. HAE-C1INH burden was assessed by disease activity (HAE-AS) and quality of life (HAE-QoL). All subjects in the study underwent CGA including Mini Mental State Examination (MMSE), Geriatric Depression Scale (GDS), Activities of Daily Living (ADLs) and Instrumental ADL (IADLs), and the Tinetti test. The comorbidity burden was measured by Charlson Comorbidity Index (CCI) and frailty status by using the Clinical Frailty Scale (CFS).

**Results:**

Thirty HAE-C1INH patients from 20 unrelated families were included and compared with age/sex matched HC (*n* = 50). HAE-AS classified disease activity as high in 20% of patients while HAE-QoL was severely impaired in 10% of patients. The CGA documented a similar distribution of MMSE, GDS, IADL, and Tinetti between patients and HC. Comorbidities were similarly represented in both the groups as well as of CCI and CFS.

**Conclusion:**

Geriatric score analysis shows that older adults with HAE-C1INH exhibit age-related frailty comparable to general population controls. As the proportion of HAE-C1INH patients achieving the geriatric age threshold is growing, defining disease-specific frailty remains crucial for enhancing care in older adults.

## 1 Background

Frailty is defined as a clinically recognizable state of increased vulnerability to stressors, resulting from multiple factors and the effects of aging on the systems’ functions (1). It is characterized by a reduced functional reserve and a diminished intrinsic capacity to cope with stressful events, exposing the individual to an increased risk of adverse outcomes, including disability, hospitalization, and even death (2). The high incidence of comorbidities, associated with potential cognitive impairment, malnutrition, reduced physical activity, and social isolation, contributes to frailty, further increasing susceptibility to *Frailty Syndrome* (FS) (3). The prevalence of FS among the global elderly population ranges between 4 and 27%, with a proportional increase related to age (4). Among the most frequent diseases in the over-65 population are arterial hypertension and cardiovascular diseases, metabolic disorders (including diabetes mellitus and dyslipidemia), chronic obstructive pulmonary disease (COPD), renal failure, neurodegenerative diseases, and other conditions associated with disability (5, 6). Geriatric frailty is, thus, conventionally associated with physiological age-dependent decline and the accumulation of damage and deficits. In rare diseases, which are often genetic and chronic, the framework shifts: the underlying disorder itself could trigger and drive the development of overall vulnerability. In this context, a rare disease acts as a chronic stressor capable of initiating a trajectory of susceptibility to FS. Therefore, risk stratification models that integrate disease-specific frailty with other geriatric indices might guide therapeutic decisions and improve outcomes for affected patients in the geriatric age group. Recently, a prospective study analyzed frailty in patients suffering from a rare connective tissue disease, Systemic Sclerosis (SSc) and documented a significant prevalence of frailty among SSc patients thus supporting the role of frailty assessment as a crucial tool for management strategies (7). Furthermore, particularly in rare diseases, frailty can be considered a key indicator of disease severity and can also help guide therapeutic decisions (8, 9). While frailty has been primarily defined as clinical and functional, it is increasingly being recognized as a multidimensional condition that also encompasses potential psychological and/or adjustment disorders, as well as social isolation.

Hereditary Angioedema due to C1-inhibitor deficiency (HAE-C1INH) is a rare, genetically determined disease with onset in childhood and a variable course to the geriatric age representing therefore a rare and lifelong condition (10). HAE-C1INH is an autosomal dominant disease: more than 500 mutations have been documented in the literature, including deletions, insertions, duplications, missense, and nonsense mutations (10). The quantitative (type I HAE) or qualitative (type II HAE) defect of C1INH causes a lack of control over the complement and fibrinolytic activation pathways, leading to elevated levels of bradykinin (BK) and thereby BK-mediated angioedema attacks. Acute attacks can involve any site, including face, upper airways, genitals, extremities, and gastrointestinal tract; angioedema attacks last up to 5 days, causing pain and functional impairment (11). In cases of clinical suspicion of HAE-C1INH, diagnosis is based on the measurement of plasma levels of C4, C1INH (both antigenic and functional), and C1q. Analysis of mutations in the SERPING1 gene is recommended, by using techniques such as Next Generation Sequencing (NGS), Multiple Ligation-dependent Probe Amplification (MLPA), and Sanger sequencing (12, 13). The unpredictability of acute attacks, the functional limitations associated with the affected sites and the related pain, the access to treatments, and the frequent need for caregivers impact social isolation and emotional burden in HAE-C1INH patients (14, 15). To date, evidence from the literature aimed at describing specific needs and diagnostic-therapeutic challenges in “special” populations such as pediatric and adolescent (16, 17) as well as pregnant women (18). However, the geriatric age represents a “special population” which is characterized by specific clinical needs and therapeutic challenges, given the presence of comorbidities and consequent polypharmacy, as well as potential concomitant disability (2).

Patients suffering from this rare and genetically determined disease, particularly in the geriatric age group, could exhibit significant vulnerability, potentially further complicated by social isolation, which would represent additional factors to age-related frailty (15). To date, there is no evidence investigating multidimensional frailty in geriatric patients affected by HAE-C1INH.

The study aimed at analyzing for the first time frailty in geriatric patients with HAE-C1INH by using Comprehensive Geriatric Assessment (CGA) scores and to compare it with the frailty documented from general geriatric population. Secondary objective was to correlate CGA scores with disease-specific scales for HAE-C1INH, such as the HAE-AS (HAE-Activity Score) and HAE-QoL (HAE-Quality of Life). Distribution of comorbidities in patients and controls has also been registered.

## 2 Patients and methods

A single-center cross-sectional comparative study with a control group included, from June to December 2025, patients with a defined diagnosis of HAE-C1INH recruited from the Reference Center for HAE at Tor Vergata University Hospital in Rome (Italy). Healthy controls (HC) were enrolled from subjects referring to the Geriatric Unit, at the same Hospital.

Inclusion criteria were: (1) a defined diagnosis of HAE-C1INH according to international criteria (19); (2) age ≥65 y.o.; (3) consent to study. Exclusion criteria for all subjects in the study were: (1) a documented severe disability; (2) malignancies within 5 y.o. and/or on active treatments; (3) documented moderate–severe cognitive impairment [defined as Mini Mental State Examination (MMSE) ≤ 18]; (4) concomitant diagnosis of neurodegenerative diseases. Patients were compared with a age and sex-matched control group. Demographic and clinical data, and therapies were registered. Records from patients included disease history and age at the disease onset/diagnosis, concomitant disorders, and therapies. From patients in the study, peripheral serum levels of complement components (C4, C1q, and C1INH—both the antigen and the functional assay) and specific SERPING1 genetic mutations were registered. C1q, C4, and C1INH (antigen) were examined by turbidimetric immunoassay while C1INH function was measured by ELISA: normal values were C4 12–36 mg/dL, C1q 110–220 mg/L, C1INH 21–38 mg/dL, and C1INH function ≥ 68%. To obtain molecular characterization of the disease, patients were referred to the Medical Genetics Unit of the same Hospital for genetic counseling and molecular analysis. During pre-test counseling, familial history and pedigrees were analyzed and informed written consent was collected. Genomic DNA was isolated from peripheral blood mononuclear cells and NGS analysis was performed with an on-demand Ion AmpliSeq™ targeted panel (Thermo Fisher Scientific) comprising all coding regions and exon/intron junctions (±20 bp) of the SERPING1 gene (NM_000062.3). Variants were described following HGVS Nomenclature and classified according to the ACMG/AMP criteria (12, 13). MLPA was performed using the SALSA MLPA Probemix P243-B1 SERPING1-F12 (MRC-Holland, Netherlands), according to the manufacturer protocol. During post-test counseling, genetic results were discussed along with familial recurrence risk and segregation analysis was recommended, when appropriate (20).

In patients with HAE-C1INH, disease assessment was performed by expert Immunologists at the Reference Center: disease activity is measured by using the HAE-AS, and QoL is evaluated via the HAE-QoL (21). According to the HAE-AS, a total raw score (12 items) ranges from 0 to 29, which is converted into a linear measure on a 0–30 scale. A raw score of ≤ 12 indicates mild or low disease activity; a raw score of ≥ 13 indicates severe disease activity. In HAE-QoL, a total score (25 items) is divided into 7 specific domain scales: physical functioning, stigma, emotional role, concern about offspring, perceived control, mental health, and treatment difficulties (22). The HAE-QoL calculates results as values from 0 to 100 for each domain [where 0 represents the worst possible quality of life in that domain (maximum disease impact) and 100 represents the best possible quality of life (no disease impact)] (22).

All participants included in the study underwent CGA at the Geriatric Unit of the Tor Vergata University Hospital in Rome (Italy), performed by expert Geriatricians. CGA is performed during outpatient geriatric clinical follow-ups scheduled by age. In all participants (patients and controls), the CGA utilizes the following tools: the Mini Mental State Examination (MMSE) for cognitive screening, the Geriatric Depression Scale (GDS) to screen for depression, Activities of Daily Living (ADLs) and Instrumental Activities of Daily Living (IADLs) to quantify functional loss, and the Tinetti Scale for fall risk. Specifically, the assessment scores are classified as follows: MMSE: severe (<12), moderate (12–18), mild (>18–24), and no cognitive impairment (>24) (23); GDS: severe (11–15), mild (6–10), and no depression 0–5 (24); ADLs: 0 to 6 for lost activities (0/6 corresponds to a fully independent subject, 6/6 to a fully dependent subject) (25); IADLs: 0 to 8 for lost activities (0/8 corresponds to a fully independent subject, 8/8 to a fully dependent subject) (26); Tinetti Scale: high (≤18), moderate (19–23), and low (≥24) fall risk (27). The disease burden was measured using the Charlson Comorbidity Index (CCI) while the frailty status using the Clinical Frailty Scale (CFS): CCI, age-adjusted score [<50 years (+0), 50–59 years (+1), 60–69 years (+2), 70–79 years (+3), ≥ 80 years (+4)]; CFS: frailty classified from 1 to 9 (very fit, well, managing well, vulnerable, mildly frail, moderately frail, severely frail, very severely frail, and terminally ill) (28, 29).

The study has been carried out according to the code of Ethics of the World Medical Association (Declaration of Helsinki) for experiments involving humans (updated 2013). Informed consent was obtained from all subjects, and the study was approved by the scientific ethic committee of the Tor Vergata University Hospital in Rome (Italy).

### 2.1 Statistical analysis

Data normality was assessed using the D’Agostino-Pearson omnibus test. Normally distributed variables are expressed as mean ± standard deviation (SD), while non-normally distributed variables are reported as median with interquartile ranges. Continuous variables were compared using the parametric unpaired *t*-test or the non-parametric Mann–Whitney U test, as appropriate. Categorical variables are presented as absolute frequencies and percentages and were compared using the Chi-squared test or Fisher’s exact test, as appropriate. Correlations were evaluated using Pearson’s correlation test. *p*-values < 0.05 were considered statistically significant. All statistical analyses were performed using GraphPad Prism version 9 (GraphPad Software, San Diego, CA, United States).

## 3 Results

### 3.1 Study population

Among 34 consecutive patients with age ≥ 65 y.o., 30 fulfilled all inclusion criteria and completed the study (Table 1). HAE-C1INH patients, from 20 unrelated families, were diagnosed with type I HAE in 90% (27/30) while the remaining 10% had type II; a positive family history of HAE-C1INH was documented in 76.7% (23/30) of patients (Table 1). Disease-causing SERPING1 variants were all present in the heterozygous state (Table 1). Mean levels of complement components at the time of the study were: C4 9.6 ± 6.7 mg/dL, C1q 185 ± 24 mg/L, C1INH 10.8 ± 6.2 mg/dL, and C1INH function 33.5 ± 11.7%. The diagnostic delay in C1INH-HAE patients was 24.4 ± 14.2 years, with no gender differences in the distribution of years.

**Table 1:** Disease-causing SERPING1 variants from C1INH-HAE patients.

ID	Sex	Age at study (yrs.)	Age at diagnosis (years)	HAE family history	HAE type	Nucleotide change	Full protein variant	ACMG class
01	F	85	47	Yes	I	c.-22-1G > A	p.?	C5
02	F	82	43	Yes	I	c.410C > A	p.(Ser137Ter)	C4^a^
03	M	79	37	Yes	I	c.410C > A	p.(Ser137Ter)	C4^a^
04	F	79	34	Yes	I	c.551-5 T > A	p.?	C5
05	M	72	30	Yes	II	c.1396C > A	p.(Arg466Ser)	C5
06	F	74	57	No	I	c.727del	p.(Leu243CysfsTer9)	C5
07	M	73	50	Yes	I	c.[998C > G; 1,004 T > G]	p.[(Ala333Gly; Phe335Cys)]	C5
08	M	97	62	Yes	I	c.[998C > G; 1,004 T > G]	p.[(Ala333Gly; Phe335Cys)]	C5
09	M	67	44	Yes	I	c.[998C > G; 1,004 T > G]	p.[(Ala333Gly; Phe335Cys)]	C5
10	F	74	31	Yes	I	c.551-5 T > A	p.?	C5
11	F	74	47	Yes	I	c.551-5 T > A	p.?	C5
12	F	72	35	Yes	I	c.551-5 T > A	p.?	C5
13	M	91	46	No	I	c.550G > A	p.(Gly184Arg)	C5
14	M	68	42	No	II	c.1396C > T	p.(Arg466Cys)	C5
15	F	70	26	Yes	I	c.551-5 T > A	p.?	C5
16	M	66	56	No	I	N/A	N/A	N/A
17	M	78	64	Yes	I	N/A	N/A	N/A
18	F	72	32	Yes	I	exon 4–8 deletion	p.?	C5
19	F	68	44	No	I	c.896G > A	p.(Trp299Ter)	C5
20	M	75	30	Yes	I	N/A	N/A	N/A
21	M	76	49	Yes	I	c.551-5 T > A	p.?	C5
22	M	86	51	Yes	I	c.1350del	p.(Glu451ArgfsTer125)	C5
23	M	72	50	No	II	c.1396C > T	p.(Arg466Cys)	C5
24	M	75	30	Yes	I	c.722G > C	p.(Arg241Pro)	C4
25	F	72	30	Yes	I	c.722G > C	p.(Arg241Pro)	C4
26	F	79	33	Yes	I	c.1129_1130insA	p.(Ser377TyrfsTer48)	C5
27	M	69	56	Yes	I	c.1480C > T	p.(Arg494Ter)	C5
28	F	78	44	Yes	I	c.1350del	p.(Glu451ArgfsTer125)	C5
29	F	84	39	Yes	I	exon 4 deletion	p.?	C5
30	F	79	34	No	I	c.164del	p.(Phe55SerfsTer24)	C5

Classification is reported according to the American College of Medical Genetics and Genomics and the Association for Molecular Pathology (ACMG/AMP): C5, pathogenic; C4, likely pathogenic (12, 13); the p.? notation means that the identified nucleotide change is expected to have an effect at the protein level. F = female; M = male; N/A = genetic test not available at the time of the study.

a Not previously described variant.

At the time of the study, 33.4% of patients (10/30) were on long term prophylaxis (LTP): half of them (*n* = 5) received subcutaneous plasma-derived C1INH and the remaining 50% (*n* = 5) received attenuated androgens at an average dosage of 80 ± 44.7 mg/day (range: min 50 mg/day – max 150 mg/day). No significant differences emerged between male and female patients regarding the distribution of positive family history, diagnostic delay, or current therapy [LTP vs. on demand therapy (ODT)].

#### 3.1.1 Disease activity and QoL in HAE-C1INH

**Figure 1:**
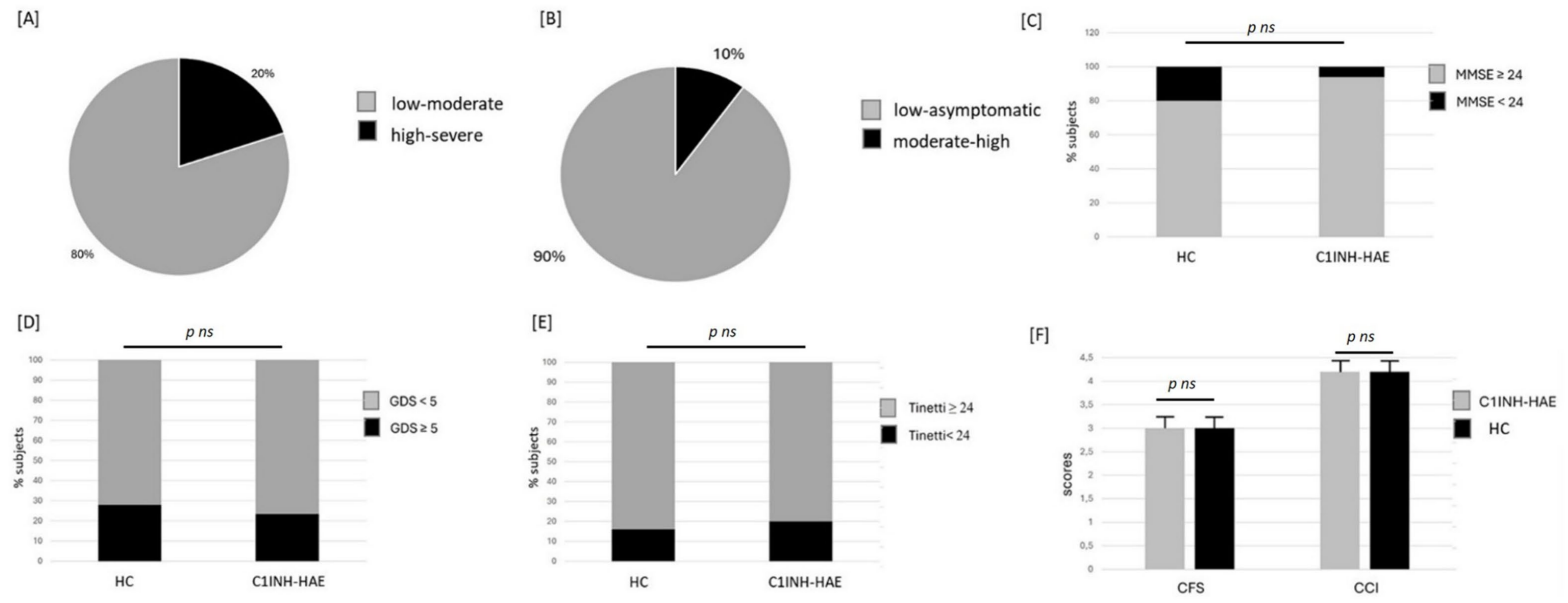
Clinical assessment in patients and controls at the time of the study. Disease activity **(A)** and quality of life **(B)** according to Hereditary Angioedema Activity Score and Hereditary Angioedema Quality of Life, respectively, in patients with HAE-C1INH. Distribution of Mini Mental State Examination (MMSE, **C**), Geriatric Depression Scale (GDS, **D**), Tinetti **(E)**, Clinical Frailty Scale (CFS) and Charlson Comorbidity Index (CCI, **F**), in patients and controls (HC). *p*-values greater than 0.05 are reported as *ns* (not significant).

All included patients completed PROs for HAE. The mean HAE-AS value recorded in the patient population was 10.3 ± 2.9 on a scale of 0–30. In 20% of cases (*n* = 6), disease activity at the time of the study was classified as high (≥13) according to the HAE-AS score (over the previous 6 months) (Figure 1A). The mean HAE-QoL value recorded in the patient population was 95.5 ± 25.5 (Figure 1B); in 10% of patients (*n* = 3), quality of life was found to be severely impaired according to HAE-QoL score (16.7 ± 4.2, mean ± SD). No statistically significant differences emerged regarding the distribution of HAE-AS and HAE-QoL scores when comparing male and female patients.

#### 3.1.2 Comorbidities from the study population

Patients were compared with 50 age and sex-matched HC (Table 2). In the patient group, *n* = 1 patient (3.4%) was professionally active, while no active workers were recorded in the control group. Smoking habits—current or former—were reported in a similar prevalence among patients (5/30, 16.7%) and controls (5/50, 10%).

**Table 2:** Data from the study cohorts.

Variables	HAE-C1INH (*N* = 30)	HC (*N* = 50)
Age at the study (mean ± SD, yrs.)	76.2 ± 7.2	78 ± 1
F:M	1	1
HAE-C1INH family history (*N*/%)	23/76.7	N/A
Type I HAE-C1INH (*N*/%)	27/90	N/A
Diagnostic delay (mean ± SD, yrs.)	24.4 ± 14.2	N/A
LTP (*N*%)	10/33.4	N/A
BMI ≥ 30 (*N*/%)	1/3.4	2/4
Hypertension (*N*/%)	21/70	41/82
Dyslipidemia (*N*/%)	17/56.7	21/42
Chronic heart diseases (*N*/%)	10/33.4	10/20
Type II diabetes (*N*/%)	4/13.4	16/32
Thyroid diseases (*N*/%)	8/26.7	6/12
Ocular pathologies (*N*/%)	3/10	13/26
Autoimmune diseases (*N*/%)	2/6.7	2/4
Malignancies (*N*/%)	2/6.7	8/16
Osteoporosis (*N*/%)	6/20	13/26
Gastrointestinal diseases (*N*/%)	7/23.4	13/26
Chronic obstructive pulmonary disease (*N*/%)	3/10	4/8
Chronic HCV infection (*N*/%)	2/6.7	0/0
Chronic kidney disease (*N*/%)	0/0	2/4

HAE-C1INH, Hereditary Angioedema due to C1-inhibitor deficiency; HC, healthy controls; LTP, Long Term Prophylaxis; BMI, Body Mass Index.

Patients with a BMI ≥ 30 were equally represented in both groups (Table 2). The most prevalent comorbidity in both patients and controls was hypertension, with similar distribution. In both the groups, the second most common condition after hypertension was dyslipidemia, showing a trend of a higher prevalence in patients compared to HC (while non-significant, P 0.2). The third most common condition in the HAE-C1INH group was ischemic heart disease, with a prevalence comparable to controls. Conversely, in the control group, the third most common condition after dyslipidemia was type II diabetes, which showed a higher, though not statistically significant, prevalence compared to HAE-C1INH patients (P 0.06). Thyroid diseases were documented more frequently in patients than in controls, though without a significant difference (P 0.09). On the other hand, ocular pathologies were recorded more often in controls than in patients (P 0.08). A similar prevalence was documented in both patients and controls for malignancies, systemic autoimmune diseases, osteoporosis, gastrointestinal diseases, and COPD. Chronic HCV infection occurred only in the patient group, while chronic kidney disease was described among controls.

#### 3.1.3 Comprehensive geriatric assessment in the study population

All included patients underwent the CGA (Table 3). The evaluation of the cognitive domain in HAE-C1INH patients documented a mean MMSE score comparable to that recorded in HC. An MMSE score ≥24 showed a higher prevalence trend in HAE-C1INH patients compared to HC (Figure 1C). No differences in MMSE scores were observed between male and female subjects. The distribution of GDS scores documented a similar proportion of subjects with a GDS ≥ 5 in both the HAE-C1INH patient group and the control group (Figure 1D). Regarding activities of daily living, subjects with more than one ADL lost at the time of the visit were significantly more prevalent in the control group compared to the HAE-C1INH group with *p* < 0.01 (Table 3). Conversely, subjects with more than one IADL lost were equally represented in the control group compared to the HAE-C1INH group (Table 3). The fall risk, evaluated using the Tinetti scale, was comparable between the two groups (Figure 1E).

**Table 3:** Comprehensive geriatric assessment from the study cohorts.

Scores	HAE-C1INH (*N* = 30)	HC (*N* = 50)
MMSE (mean ± DS)	27.4 ± 2.7	26.3 ± 3.3
GDS ≥ 5 (*N*/%)	6/20	14/28
ADL ≥ 1 (*N*/%)	1/3.4*	13/26
IADL ≥1 (*N*/%)	8/26.7	9/18
Tinetti ≥24 (*N*/%)	25/83.4	40/80
CCI (mean ± SD)	4.2 ± 0.9	4.2 ± 1.2
CFS (mean ± SD)	3 ± 0.9	3.2 ± 0.8

HAE-C1INH, Hereditary Angioedema due to C1-inhibitor deficiency; HC, Healthy Controls; MMSE, Mini Mental State Examination; GDS, Geriatric Depression Scale (GDS ≥5 as a cut-off for depression); ADL, Activities of Daily Living; IADL, Instrumental Activities of Daily Living; Tinetti ≥24 as a cut-off for low fall risk; CCI, Charlson Comorbidity Index; CFS, Clinical Frailty Scale. * *p* = 0.01.

The overall disease burden in the study subjects, assessed according to the CCI was similar in HAE-C1INH patients and controls (Figure 1F). Likewise, the level of frailty documented in the study population according to the CFS was similar in both patients and controls (Figure 1F).

In the HAE-C1INH patient population, no significant correlations were found between MMSE or GDS scores and the HAE-AS or HAE-QoL values. Furthermore, no correlations were observed between CCI values and HAE-AS or HAE-QoL, nor between CFS values and HAE-AS or HAE-QoL scores.

## 4 Discussion

The present study provides, for the first time, an analysis of the multidimensional geriatric frailty condition in patients affected by a rare disease, HAE-C1INH, describing a comparison between these rare patients and subjects representative of the general geriatric population. This research study was conducted by exploring functional, cognitive, and psychological domains within the context of a validated CGA, widely used in both research studies and clinical practice. The present cross-sectional study with an exploratory purpose is the first in the literature to analyze frailty in geriatric patients with HAE-C1INH, contributing to the understanding of this rare disease according to the progressive aging of the general population—and, consequently, of patients living with rare genetic diseases.

To date, evidence from the literature focus on HAE-C1INH disease phenotypes in pediatric and adolescent populations, along with findings from case series and cohort studies on pregnant women. All these studies mainly aim at defining specific needs and diagnostic-therapeutic challenges in these “special” populations (16–18). However, geriatric patients represent a special population too, which characterized by specific clinical and therapeutic needs, as well as management challenges due to the presence of comorbidities, polypharmacy, and age-related changes in hepatic metabolism and/or renal function (2). In women with HAE-C1INH after menopause, the disease activity seems to remain stable in most cases; however, women who exhibit estrogen sensitivity during their childbearing years tend to experience significant improvement in the course of the disease post-menopause (30). Nevertheless, to date, there is no evidence regarding a potential correlation between the severity of climacteric symptoms and the HAE disease burden, nor between specific comorbidities and the disease course after menopause (30). In this context, comorbidities, which lead to polypharmacy—and consequently to increased risk of drug-induced disorders and interactions—complicate the quality of life for geriatric patients. In this age group, the effects of concomitant conditions increase the burden of the rare disease, thus potentially contributing to patient vulnerability (7, 31). Additionally, the lack of effective HAE-C1INH treatments in the past could affect significantly the lifelong disease burden of the current geriatric cohort. As well reported, frailty is a progressive, multifactorial, and ever-increasing condition leading to decline of multiple systems and, thus, a reduced ability to respond to stress (1, 2). The assessment of frailty in geriatric patients is crucial for the early identification of subjects at risk of functional decline (“pre-frailty”), allowing for the adoption of targeted preventive strategies (32). In geriatric patients with HAE-C1INH, frailty can be further exacerbated by the disease course characterized by unpredictable, debilitating, and potentially fatal acute attacks. The anxiety for acute attacks and the related social isolation are well-reported complications for patients with HAE-C1INH who, in the geriatric stage, are *per se* characterized by a tendency toward social isolation and depression (33–35). Furthermore, HAE-C1INH is a lifelong diagnosis requiring long-term follow-up and treatment that remain mainly invasive, often poorly tolerated, and variable in efficacy. For these reasons, including frailty assessment into the clinical management of HAE-C1INH patients could serve as a valuable tool for selecting the most appropriate care and therapeutic strategies to improve quality of life and optimize the allocation of healthcare resources. Findings from our original study document a diagnostic delay significantly longer than that is currently reported in the literature from the overall HAE-C1INH patients: it could be related to the enrolled population who received HAE-C1INH diagnosis during an era when disease awareness and diagnostic tools were markedly limited (19). Therefore, results from this study are consistent with the era of symptom onset for patients who are currently in the geriatric age group (15). Comorbidities show a comparable distribution in the patient group relative to the control group, except for dyslipidemia which showed a trend to be greater in HAE-C1INH patients probably linked to the past and/or current use of attenuated androgens which served as a first-line treatment, particularly in the era before the availability of plasma-derived C1 inhibitor (15). Using specific PROs, the patients included in the study report satisfactory disease control, and the angioedema-related quality of life results significantly compromised only in a small number of cases. This is consistent with the data recorded from the multidimensional assessment, particularly regarding the psychological domain: in fact, the results show a similar proportion of subjects with depression (according to the GDS) in both controls and patients. These data might describe that, when the disease is well-controlled, its expected emotional impact could be reduced. It is noteworthy that one-third of the patient cohort is on LTP at the time of the study, thereby maintaining continuous control over attack recurrence. The explored functional domains (ADL, IADL, Tinetti) reveal that geriatric patients with HAE-C1INH are clinically robust from both the functional and the mobility status. Specifically, they are fully independent in both basic and instrumental activities of daily living and do not show an increased risk of falls.

However, geriatric frailty in rare diseases includes additional considerations since the underlying disease could trigger the overall vulnerability. Although autonomy and function result normal according the standard frailty tools, it might be crucial to identify a disease-specific frailty in HAE-C1INH. Focusing on frailty is a current trend in rare diseases because the patient population is aging: authors reported in elderly patients with rare immune-rheumatological disorders a higher prevalence of vulnerability highlighting how olders face unique challenges and require a tailored approach (7, 8, 36).

Main limitations of this study are the sample size and the potential selection bias of the included cohort: given the relatively small sample size, analyses are primarily descriptive and no subgroup comparisons or multivariate analyses have been adequately performed. Thus, non-significant results could be merely a consequence of an underpowered study. Nevertheless, the sample size could be representative given the rarity of the disease. Moreover, given its exploratory nature, this study aims to provide a descriptive map of a timely question that remains largely undocumented and serves at establishing an empirical basis required for subsequent analyses. The potential selection bias arising from the inclusion of patients referring to a specialized reference center may limit the generalizability of the findings to the broader HAE-C1INH population. However, the single-center approach is advantageous as it allows the same team of Researchers to conduct all immunological and geriatric assessments, effectively minimizing inter-operator variability.

Comparing the patient cohort with the geriatric controls by using the standard CGA as a frailty instrument allows to document that HAE-C1INH patients do not have increased frailty levels in the geriatric stage. However, elderly patients with this genetic disease represent a distinct target population thus requiring tailored tools to capture additional and disease-specific frailty domains. A useful strategy in clinical practice caring elderly HAE-C1INH patients could be the implementation of comprehensive geriatric assessment in the prospective follow-up to detect emerging frailty at its onset. This approach can guide therapeutic choices and patient management, including the requirement for a caregiver and the necessity of multidisciplinary care.

## 5 Conclusion

The present study evaluates, for the first time, geriatric frailty in patients affected by HAE-C1INH, documenting a strong biological and functional reserve from HAE-C1INH patients with a similar distribution of comorbidities with respect to the general geriatric population. Cognitive and psychological domains appear to remain maintained in the presence of a good disease control with preserved quality of life. We might hypothesize that the classical frailty tools may miss relevant vulnerability in HAE-C1INH patients thereby introducing a future perspective to explore this field. Even though this hypothesis is not directly supported by findings from the study, it could be suggested according to the disease burden, which is life-long and affected by numerous challenges potentially affecting the multidimensional geriatric frailty.

Given the progressive aging of the population and the increasing availability of novel therapies, an age-specific approach is both expected and necessary to improve outcomes, enhance quality of life, and optimize the allocation of healthcare resources.

## Data Availability

The raw data supporting the conclusions of this article will be made available by the authors, without undue reservation.
